# The Phosphodiesterase 4 Inhibitor Roflumilast Protects Microvascular Endothelial Cells from Irradiation-Induced Dysfunctions

**DOI:** 10.3390/cells14131017

**Published:** 2025-07-03

**Authors:** Nathalie Guitard, Florent Raffin, François-Xavier Boittin

**Affiliations:** 1 Unité de Radiobiologie, Département Effets Biologiques des Rayonnements, IRBA (Institut de Recherche Biomédicale des Armées), 91223 Brétigny-sur-Orge, France; 2 Unité Analyses Biologiques, Département des Plateformes, IRBA (Institut de Recherche Biomédicale des Armées), 91223 Brétigny-sur-Orge, France

**Keywords:** endothelial cells, VE-Cadherin, actin cytoskeleton, irradiation, apoptosis, ICAM-1

## Abstract

In endothelial cells, high-dose irradiation induces numerous dysfunctions including alteration in junctional proteins such as VE-Cadherin, apoptosis and enhanced adhesiveness linked to overexpression of adhesion molecules like Intercellular Adhesion Molecule 1 (ICAM-1). Such endothelial dysfunctions can lead to altered tissue perfusion, development of tissue inflammation through increased endothelial permeability, and ultimately organ damage. As intracellular cyclic AMP (cAMP) levels are known to control intercellular junctions or apoptosis in the endothelium, we investigated here the effect of the Phosphodiesterase 4 inhibitor Roflumilast, a drug increasing cAMP levels, on irradiation-induced endothelial dysfunctions in human pulmonary microvascular endothelial cells (HPMECs). Using continuous impedance measurements in confluent endothelial cell monolayers, Roflumilast was found to rapidly reinforce the endothelial barrier and to prevent irradiation-induced barrier disruption. In accordance, irradiation-induced alteration in membrane VE-Cadherin-composed adherens junctions was prevented by Roflumilast treatment after irradiation, which was correlated with its protective effect of the actin cytoskeleton. Post-irradiation treatment with Roflumilast also protected HPMECs from irradiation-induced late apoptosis, but was without effect on irradiation-induced ICAM-1 overexpression. Overall, our results indicate that the beneficial effects of Roflumilast after irradiation are linked to the strengthening/protection of the endothelial barrier and reduced apoptosis, suggesting that this medicine may be useful for the treatment of endothelial damages after exposure to a high dose of radiation.

## 1. Introduction

Dysfunctions of the blood vessel endothelium are involved in the development of tissue/organ damages following exposure to ionizing rays [1]. High-dose irradiation can indeed trigger overexpression of adhesion molecules, apoptosis and alteration in cell junctions in endothelial monolayers, which induces exacerbated leucocyte infiltration and inflammation, tissue oedema, and impaired tissue/organ perfusion [1,2,3,4,5,6,7,8,9].

The endothelial barrier of peripheral vessels is mainly regulated by the assembly of the adhesive protein VE-Cadherin, which connects adjacent endothelial cells through its extracellular cadherin domains [10,11,12,13,14]. Endothelial permeability can be regulated by the phosphorylation and internalization of VE-Cadherin and its associated proteins, but also by the actin cytoskeleton [10,11,12,13,14]. Indeed, the formation of radial cytosolic actin stress fibers can exert tension on adherens junction, which can trigger cell contraction/retraction, formation of discontinuous/focal adherens junction and gaps between endothelial cells [10,11,12,13,14]. Stress fiber formation can be triggered by agents increasing endothelial permeability (Vascular Endothelial Growth Factor, Histamin, Thrombin, etc.) and is linked to the activation of the small GTPase RhoA/Rock pathway, which indeed stimulates myosin activation and actin polymerization [10,11,12,13,14].

Irradiation of endothelial cells has also been shown to increase endothelial permeability and to alter plasma membrane adherens junctions/VE-Cadherin [5,9,15,16,17,18]. This effect has been shown to be in part related to the activation of the RhoA/Rock pathway, leading to actin stress fiber formation, cell contraction, adherens junction alteration/opening and formation of gaps between cells in the monolayer [17,19]. However, several studies also indicate that irradiation-induced permeability increase is linked to the degradation of VE-Cadherin by proteolytic enzyme such as ADAM10 (A Disintegrin And Metalloproteinase 10) or Calpains and its internalization toward intracellular compartments [5,15,18]. Finally, irradiation-induced apoptosis of endothelial cells is also believed to contribute to the permeability increase observed after irradiation [2,8,20].

cAMP has long been demonstrated to be an important regulator of endothelial cell adhesion and permeability [10,11,12,21,22,23]. cAMP enhances endothelial barrier function by facilitating the formation of stable VE-Cadherin adherens junctions between endothelial cells through multiple pathways [10,11,12,21,22,23]. Indeed, cAMP inhibits the RhoA-Rock pathway, which reduces the formation of stress fibers and focal adherens junctions [10,11,12,22]. In contrast, cAMP promotes the formation of circumferential actin and stable/linear adherens junction through the CDC42 pathway [10,22]. Finally, cAMP levels can control VE-Cadherin expression through a pathway involving the activation of the CREB transcription factor [24].

In addition to its effects on the cytoskeleton and adherens junctions, cAMP and its signaling pathways have been described as regulators of apoptosis in numerous cell types including endothelial cells [25,26]. In fact, cAMP can trigger apoptosis through a mitochondria-dependent pathway involving Protein Kinase A (PKA) activation, while it can prevent apoptosis through the activation of the Exchange protein activated by cAMP (Epac), a guanine nucleotide exchange factor [25,26].

The beneficial effect of cAMP on endothelial junctions may be exploited for treatment of pathological states involving increased endothelial permeability. Indeed, agents increasing cAMP levels, such as Phosphodiesterase 4 (PDE4) inhibitors, have been shown to be efficient in reducing the increased endothelial permeability occurring in animal models of sepsis or ischemia [24,27,28,29,30,31,32].

However, the effect of drugs increasing cAMP has not been investigated so far to prevent irradiation-induced endothelial dysfunctions. Here, we investigated the effect of Roflumilast, a PDE4 inhibitor increasing cAMP levels, on a panel of irradiation-induced endothelial dysfunctions in human pulmonary microvascular endothelial cells (HPMECs). Overall, we demonstrate that this medicine, already used for the treatment of chronic obstructive pulmonary disease or psoriasis, is effective to protect the endothelium from irradiation-induced endothelial dysfunctions, suggesting that it may be useful for vascular protection in case of exposure to high doses of radiation [33,34].

## 2. Materials and Methods

### 2.1. Reagents and Antibodies

Roflumilast (PDE4 inhibitor) and Phalloidin-TRITC were purchased from Tocris (Bristol, UK). The mouse monoclonal anti-VE-Cadherin (CD144) antibody (MAB9381) and the anti-mouse IgG Northerlight^TM^NL493-conjugated antibody (NL009) were from R&D systems (Minneapolis, MN, USA). The mounting medium (Fluoroshield with DAPI) was purchased from Sigma (Saint-Louis, MO, USA). For flow cytometry analysis, all antibodies (BB515 mouse anti-human CD54 (ICAM-1, cat n° 564685), BUV737 mouse anti-human CD106 (VCAM-1, cat n° 565418), PE mouse anti-human CD62P (P-Selectin, cat n° 555524), APC mouse anti-human CD62E (E-Selectin, cat n° 551144)) and the FITC Annexin V/Propidium Iodide apoptosis detection kit were obtained from BD Biosciences (Franklin Lakes, NJ, USA). The cAMP assay kit (ab290713) was from Abcam (Waltham, MA, USA), while the BCA kit was from Life technologies (Carlsbad, CA, USA).

### 2.2. Culture, Irradiation and Treatment of Microvascular Endothelial Cells

Human pulmonary microvascular endothelial cells (HPMECs, Promocell (Heidelberg, Germany)) were cultured in T25 flasks, 6-well plates or 8-chamber Lab-Tek coated with attachment factor (Gibco, Waltham, MA, USA). For impedance measurement with the xCELLigence cell analyzer, HPMECs were cultured in E-plates L16 (Acea Biosciences, San Diego, CA, USA) without coating. Endothelial cell growth medium MV2 (Promocell) supplemented with endothelial cell growth medium supplement MV2 (Promocell) and Gentamicin/amphotericin (Gibco) was used to cultivate HPMECs. As HPMECs are primary endothelial cells, confluent monolayers from passage 3 to 7 were only used in experiments. Between passages 3 and 7, endothelial cell responsiveness remained poorly variable, while HPMECs cultivated over passage 7 may exhibit significant higher proportion of apoptotic cells and altered responsiveness, preventing their use in experiments.

A SARRP X-irradiator (Xstrahl, Walsall, UK) was used for irradiation of confluent HPMECs cultured either in 6-well plates, 8-chamber Lab-Tek slides or E-plates L16 (Acea Biosciences). HPMECs were irradiated at a dose of 15 Gy, with a dose-rate value of 0.68 Gy/min. Dosimetry was performed as previously described [20,35]. In all experiments, Roflumilast was applied after irradiation (30 min).

### 2.3. Adherens Junctions and Actin Cytoskeleton Stainings

Confluent HPMECs cultured in 8-chamber Lab-Tek were washed with PBS, fixed in PBS containing 3% paraformaldehyde for 15 min, permeabilized with Triton X-100 (0.2%) for 5 min at room temperature and then incubated in PBS containing 5% bovine serum albumin (1 h at room temperature), to prevent non-specific labeling. For adherens junction staining, HPMECs were incubated in PBS containing 5% of bovine serum albumin and a mouse monoclonal anti-VE-Cadherin (CD144) antibody (diluted 1/1000) for 5 h at room temperature. After extensive washing with PBS, the cells were incubated in PBS containing 5% bovine serum albumin and an anti-mouse IgG Northerlight^TM^NL493-conjugated antibody (diluted 1/200) for 1 h at room temperature. HPMECs were then extensively washed with PBS. F-actin staining was performed by incubating HPMECs in PBS containing 1% bovine serum albumin and Phalloidin-TRITC (1/40) for 30 min at room temperature. After extensive washing with PBS, HPMECs were mounted using coverslips in Fluoroshield with DAPI (Sigma).

An Olympus BX43 microscope was used to acquire fluorescence images. Acquisition settings were kept identical to allow for a reliable comparison between the groups. To measure VE-Cadherin or F-Actin distribution (membrane or cytosol), the ratio of fluorescence intensities at the plasma membrane to the fluorescence intensities in the cytosol was calculated from the fluorescence profiles of VE-Cadherin or F-actin staining in endothelial cells (generated by image J 1.50b software). Measurements were performed on 10 fluorescence profiles from 5 representative images in each condition for each individual experiment. The proportion of cells exhibiting long stress fibers was measured in 5 representative images in each condition for each individual experiment.

### 2.4. Apoptosis Assay

As previously described, a FITC Annexin V/Propidium Iodide apoptosis detection kit (BD Biosciences) was used to measure the apoptosis of HPMECs 72 h after irradiation [20,35]. HPMECs apoptosis was measured using a Beckman Coulter Cytoflex LX cytometer (Beckman Coulter, Brea, CA, USA). FlowJo V10 software was used to perform analysis, allowing for the measurement of the proportion of apoptotic cells (PI+, FITC Annexin V+).

### 2.5. Measurements of Endothelial Cell Surface Expression of Adhesion Molecules

Membrane expression of adhesion molecules (ICAM-1, VCAM-1, P-selectin and E-selectin) was measured in HPMECs using flow cytometry, as previously described [20,35]. FlowJo software was used to measure Median Fluorescence Intensity (MFI) for ICAM-1, VCAM-1, P-selectin and E-selectin, allowing for a comparison of the cell surface expression of ICAM-1, P-selectin, E-selectin and VCAM-1 in the controls and after cell irradiation, with or without Roflumilast treatment.

### 2.6. Measurements of cAMP Concentration in Cell Lysate Samples

A competitive ELISA kit (Abcam ab290713) was used to measure cAMP concentration in cell lysate samples. HPMECs were cultured in six-well plates and then irradiated and/or treated with Roflumilast (10 µM). The samples were prepared using 0.1 M HCL, according to the manufacturer’s instructions. cAMP concentrations in samples were normalized to the total protein content measured using a BCA kit (Life Technologies). Measurements were performed 2 h after irradiation.

### 2.7. Measurements of Impedance in HPMEC Monolayers

A RTCA xCELLigence cell analyzer together with E-plates L16 (Acea Biosciences) was used to continuously monitor the dimensionless parameter cell index (proportional to impedance) of HPMEC monolayers. The cell index can be influenced by several parameters, including cell adhesion and cell growth (after seeding the cells), cell morphology and the strength of intercellular junctions. Once confluency is reached, the cell index/impedance measurements allow for an evaluation of the evolution of the endothelial barrier over time. To record cell index/impedance, HPMECs were seeded in E-plates L16 (5000 cells/well) and placed at 37 °C/5% CO_2_ in the RTCA xCELLigence cell analyzer. HPMECs were irradiated at high doses (15 Gy) and/or treated with Roflumilast only once confluence was reached (stable cell index). Cell index measurements were performed in duplicate in each experiment. Average normalized cell index values are represented at different times after cell irradiation or Roflumilast addition. To compare the rates of cell index decrease in irradiated monolayers treated or not with Roflumilast (100 nM), a linear regression analysis of normalized cell index data was performed from 6 to 24 h after irradiation and from 24 to 72 h after irradiation, allowing for the measurement of the cell index decrease per hour (slope) both early and later during experiments. GraphPad Prism 5 software (San Diego, CA, USA) was used to perform the linear regression analysis.

### 2.8. Statistical Analysis

Data are expressed as means +/− SEM and statistical analyses were performed using either *t*-tests or ANOVA tests with GraphPad Prism 5 software. The statistical significance and the number of experiments performed are indicated in the figure legends. Correlation analysis was also performed using GraphPad Prism 5 software.

## 3. Results

### 3.1. Effects of Roflumilast and Irradiation on Global cAMP Levels in HPMECs

**Figure 1:**
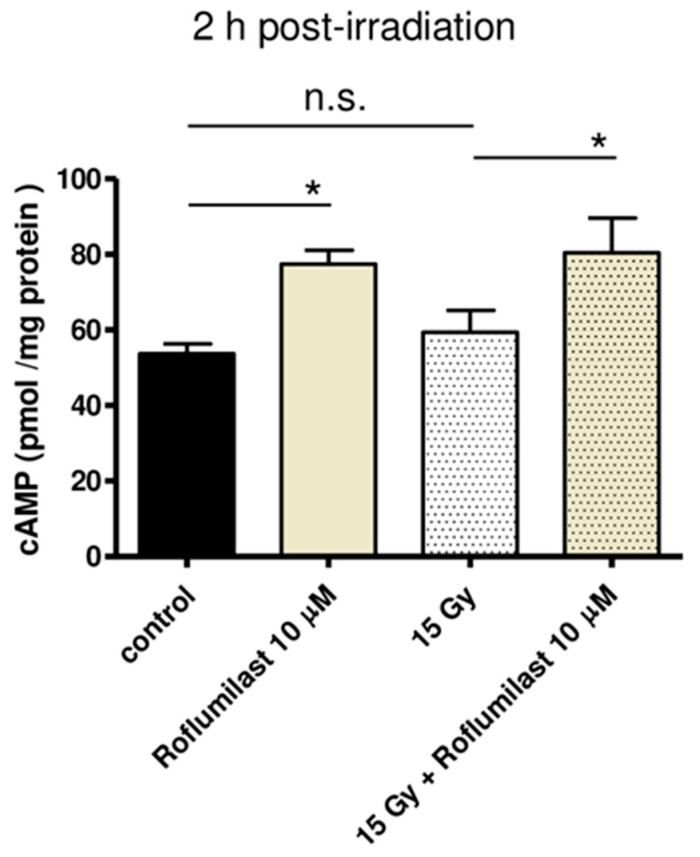
Effect of Roflumilast on cAMP concentration in control (non-irradiated) and high-dose (15 Gy)-irradiated HPMECs. Cell lysis was performed 2 h after irradiation. Global cAMP concentration was measured in cell lysates prepared from control and irradiated HPMECs treated or not with Roflumilast (10 µM) after irradiation. Data on graph represent the average of data from three experiments (* *p* < 0.05, n.s.: not significant).

cAMP concentration was measured in cell lysates using a competitive ELISA kit. As illustrated in Figure 1, Roflumilast treatment (10 µM) significantly increased cAMP concentration in HPMECs, whether cells were irradiated (15 Gy) or not. No significant difference was found between global cAMP levels of control and irradiated cells 2 h after irradiation (Figure 1).

### 3.2. Effects of Roflumilast and Irradiation on Impedance of HPMEC Monolayers

**Figure 2:**
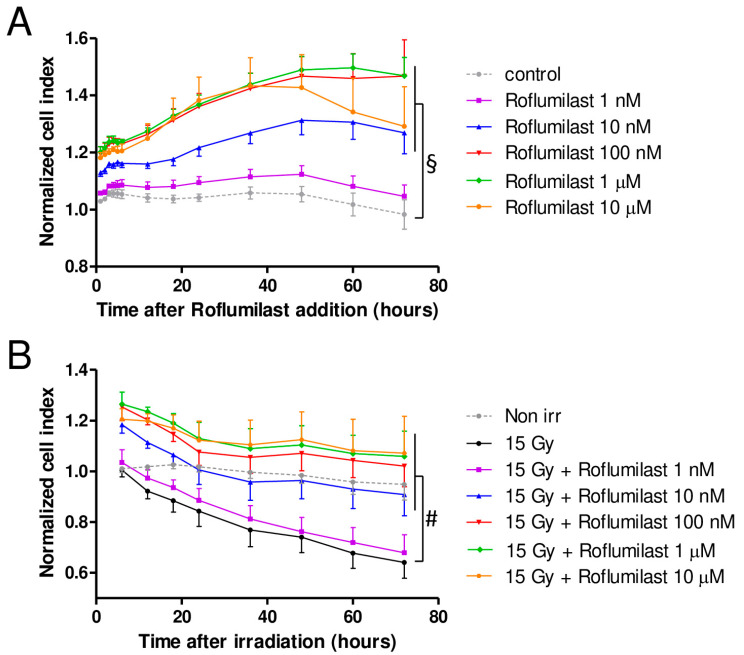

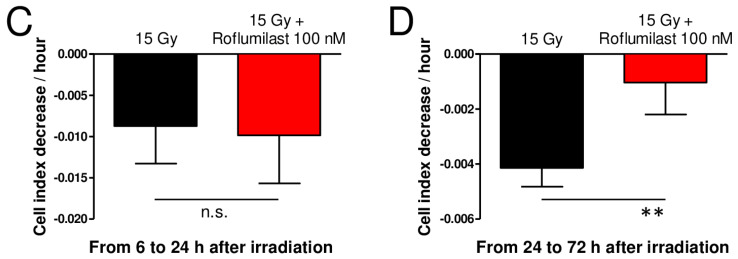
Effect of Roflumilast and irradiation on cell index/impedance of confluent HPMEC monolayers. (**A**) Average cell index values in HPMEC monolayers from 1 to 72 h after treatment with Roflumilast (1 nM, 10 nM, 100 nM, 1 µM and 10 µM). Average cell index values of non-treated HPMEC monolayers (control) are also represented. Data on graph represent average values from five experiments. (**B**) Average cell index values between 6 and 72 h after irradiation in irradiated HPMECs monolayers treated or not with Roflumilast (1 nM, 10 nM, 100 nM, 1 µM and 10 µM) after irradiation. Average cell index values of non-irradiated HPMECs monolayers (control) are also represented. Data on graph represent average values from five experiments. §: Average values for Roflumilast (≥10 nM)-treated HPMEC monolayers are significantly higher than those of the control from 1 to 72 h after Roflumilast addition (from *p* < 0.05 to *p* < 0.001 depending on time after Roflumilast addition). #: Average values for 15 Gy + Roflumilast (≥10 nM) are significantly higher than the 15 Gy average values from 6 to 72 h after irradiation (from *p* < 0.05 to *p* < 0.001 depending on time after irradiation). (**C**) Average cell index decrease per hour (slope) calculated owing to linear regression of normalized cell index data (15 Gy and 15 Gy + Roflumilast 100 nM) from 6 to 24 h after irradiation (n.s.: not significant). (**D**) Average cell index decrease per hour (slope) calculated owing to linear regression of normalized cell index data (15 Gy and 15 Gy + Roflumilast 100 nM) from 24 to 72 h after irradiation (** *p* < 0.01).

A xCELLigence cell analyzer was used to follow the impedance of confluent HPMEC monolayers. Roflumilast treatment of confluent HPMEC monolayers induced rapid and sustained increases in impedance (cell index) lasting at least 72 h, indicating that Roflumilast reinforces the endothelial barrier (Figure 2A). This effect of Roflumilast was dose-dependent and maximal for a Roflumilast concentration of 100 nM (Figure 2A). A late impedance fall was observed for HPMEC monolayers treated with 10 µM Roflumilast (36 h after Roflumilast treatment), suggesting that this high concentration of the PDE4 inhibitors may be slightly toxic for HPMECs (Figure 2A). Irradiation of HPMEC monolayers at a high dose (15 Gy) induced a slow and steady decrease in impedance starting ~6 h after the irradiation of HPMECs, corresponding to a progressive disruption of the endothelial barrier (Figure 2B). In irradiated HPMEC monolayers treated with Roflumilast at a concentration ≥10 nM, the average impedance values measured between 6 and 72 h after irradiation were significantly increased compared to monolayers that were solely irradiated, which may be related to the Roflumilast-induced impedance increase observed in non-irradiated cells, and to a protective effect of Roflumilast on irradiation-induced barrier alteration (Figure 2A). To investigate an eventual protective effect of Roflumilast on irradiation-induced barrier disruption, the rates of impedance decrease were measured both early (from 6 to 24 h after irradiation) and later (from 24 to 72 h after irradiation) in irradiated monolayers treated or not with Roflumilast (100 nM). From 6 to 24 h after irradiation, the rates of impedance decrease (cell index decrease per hour) were similar in Roflumilast-treated irradiated monolayers when compared to irradiated cells (Figure 2C). In contrast, from 24 to 72 h after irradiation, the rate of impedance decrease was significantly reduced in Roflumilast-treated endothelial monolayers when compared to monolayers that had been only irradiated, indicating that Roflumilast prevents irradiation-induced endothelial barrier disruption from 24 to 72 h after irradiation (Figure 2D).

### 3.3. Effect of Roflumilast and Irradiation on Adherens Junction in HPMEC Monolayers

**Figure 3:**
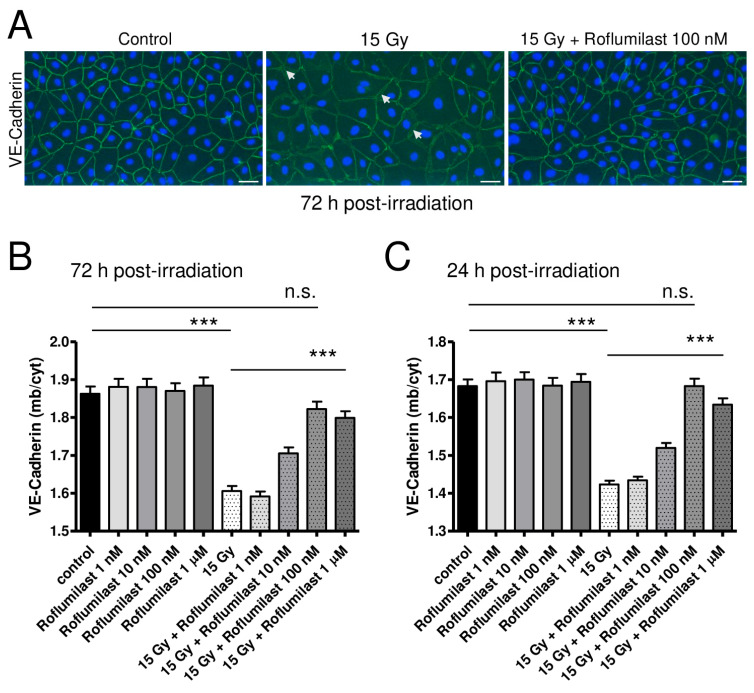
Effects of irradiation and Roflumilast on adherens junctions (VE-Cadherin) in confluent endothelial monolayers. (**A**) Stainings of VE-Cadherin (green) and nucleus (blue, DAPI) in HPMEC monolayers in control (non-irradiated cells) and 72 h after high-dose irradiation (15 Gy), with or without post-irradiation treatment with Roflumilast (100 nM). Images are representative of three independent experiments. White arrows indicate small intercellular gaps. Scale bar: 50 µM. (**B**) VE-Cadherin distribution (ratio of VE-Cadherin staining fluorescence intensities between the plasma membrane and the cytosol) in control and high-dose (15 Gy) irradiated endothelial monolayers treated or not with Roflumilast (from 1 nM to 1 µM) after irradiation. Measurements were performed 72 h after irradiation. Data on graph represent the average of measurements collected from three experiments (*** *p* < 0.001, n.s.: not significant). (**C**) VE-Cadherin distribution in control and irradiated endothelial cells (treated or not with Roflumilast) 24 h after irradiation. Data on graph represent the average of measurements collected from three experiments (*** *p* < 0.001, n.s.: not significant).

To investigate the effects of high-dose irradiation and Roflumilast on adherens junctions, confluent HPMEC monolayers were stained with an anti-VE-Cadherin antibody 72 h after irradiation. As illustrated in Figure 3A, high-dose irradiation induced obvious alterations in VE-cadherin staining in HPMECs when compared to non-irradiated cells. While VE-cadherin staining at the plasma membrane was rather regular and bright in non-irradiated cells, it appeared weaker in high-dose-irradiated HPMECs, with numerous small gaps observed between disconnected cells of the monolayer (white arrows, Figure 3A). As a consequence, the ratio between plasma membrane and cytosol VE-Cadherin staining intensities was found to be significantly reduced in irradiated endothelial monolayers (Figure 3B). This indicates that plasma membrane expression of VE-Cadherin is decreased in HPMECs 72 h after irradiation, which may be related to its degradation or its displacement toward the cytosol of endothelial cells (Figure 3B) [5,15,18]. Notably, post-irradiation treatment with Roflumilast dose-dependently restored the plasma membrane expression of VE-Cadherin, as attested by the dose-dependent recovery of the ratio between plasma membrane and cytosol VE-Cadherin staining intensities in Roflumilast-treated endothelial monolayers (Figure 3B). Therefore, Roflumilast may either protect VE-Cadherin-composed adherens junctions or facilitate their reconstitution after irradiation. In non-irradiated cells, Roflumilast did not significantly alter the plasma membrane expression of VE-Cadherin (Figure 3B).

After exposure to X-rays, an alteration in adherens junctions/VE-Cadherin was also observed sooner (24 h) after irradiation and the same protecting effect was observed after post-irradiation treatments with Roflumilast (Figure 3C). Altogether, this indicates that irradiation damages endothelial adherens junctions, and also that Roflumilast remains efficient to protect adherens junctions several days after irradiation.

As a link between the actin cytoskeleton state and VE-Cadherin/adherens junction integrity has been established, we investigated the effect of Roflumilast on actin cystoskeleton changes observed after irradiation [10,11,12,13,14].

### 3.4. Effect of Roflumilast and Irradiation on the Actin Cytoskeleton in HPMEC Monolayers

**Figure 4:**
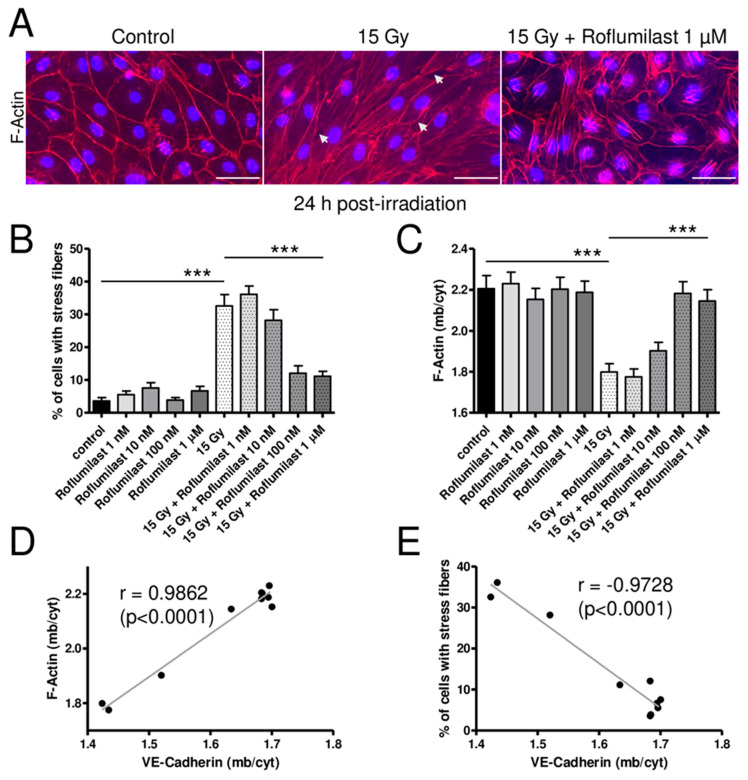
Effects of irradiation and Roflumilast on the actin cytoskeleton in confluent endothelial monolayers. (**A**) Stainings of F-actin (red) and nucleus (DAPI) in HPMECs monolayers in control (non-irradiated cells) and 24 h after irradiation at high dose (15 Gy), with or without post-irradiation treatment with Roflumilast (1 µM). Images are representative of three independent experiments. White arrows indicate small intercellular gaps. Scale bar: 50 µM. (**B**) Proportion of cells exhibiting stress fibers in control and high-dose-irradiated endothelial monolayers (15 Gy) treated or not with Roflumilast (from 1 nM to 1 µM) after irradiation. Measurements were performed 24 h after irradiation. Data on graph represent the average of measurements collected from three experiments (*** *p* < 0.001). (**C**) Distribution of F-actin (membrane/cytosol) in control and high-dose-irradiated endothelial monolayers (15 Gy) treated or not with Roflumilast (from 1 nM to 1 µM) after irradiation. Measurements were performed 24 h after irradiation. Data on graph represent the average of measurements collected from three experiments (*** *p* < 0.001). (**D**) Correlation plot of F-actin distribution (F-actin (mb/cyt)) and VE-Cadherin distribution (VE-Cadherin (mb/cyt)). (**E**) Correlation plot of the % of cells with stress fibers and VE-Cadherin distribution (VE-Cadherin (mb/cyt)).

As previously reported, high-dose irradiation (15 Gy) induced a rearrangement of the actin cytoskeleton (Figure 4) [17]. In non-irradiated HPMECs, the actin cytoskeleton was mainly located near the plasma membrane (cortical actin) but short actin fibers were sometimes observed in the nucleus area of a few cells (Figure 4A). While long actin stress fibers were rare in non-irradiated cells, they were observed in the cytosol of numerous cells in high-dose-irradiated HPMEC monolayers (Figure 4A). As a consequence, the % of cells exhibiting long stress fibers was significantly increased in irradiated monolayers 24 h after irradiation (Figure 4B). Post-irradiation treatment with high concentrations of Roflumilast (≥100 nM) significantly reduced the number of cells exhibiting long cytosolic stress fibers, while lower concentrations (1 and 10 nM) did not prevent the effect of irradiation on the actin cytoskeleton (Figure 4B). Regardless of the concentration, Roflumilast treatment alone did not significantly affect the proportion of cells exhibiting long cytosolic actin stress fibers (Figure 4B).

As indicated by white arrows on images from Figure 3A and Figure 4A, high-dose irradiation induced the formation of numerous small intercellular gaps in HPMECs monolayers. Post-irradiation treatments with Roflumilast (≥100 nM) prevented the formation of intercellular gaps in irradiated monolayers, while they were still observed in irradiated monolayers treated with a low Roflumilast concentration (1 nM).

Another obvious effect of irradiation was the significant alteration in the cortical actin staining, as evidenced by cortical F-actin distribution measurements presented in Figure 4C. Interestingly, post-irradiation treatment with Roflumilast (≥100 nM) prevented this deleterious effect of irradiation, as indicated by the similar average values of cortical actin distribution in control and irradiated endothelial cells treated with Roflumilast. A strong positive correlation (r = 0.9862) was found between the distribution of VE-Cadherin and cortical actin in control and irradiated HPMECs treated or not with increasing concentrations of Roflumilast (Figure 4D). In contrast, a strong negative correlation (r = −0.9728) was observed between the distribution of VE-Cadherin and the % of cells with long stress fibers (Figure 4E).

### 3.5. Effect of Roflumilast on Irradiation-Induced Apoptosis of HPMECs

**Figure 5:**
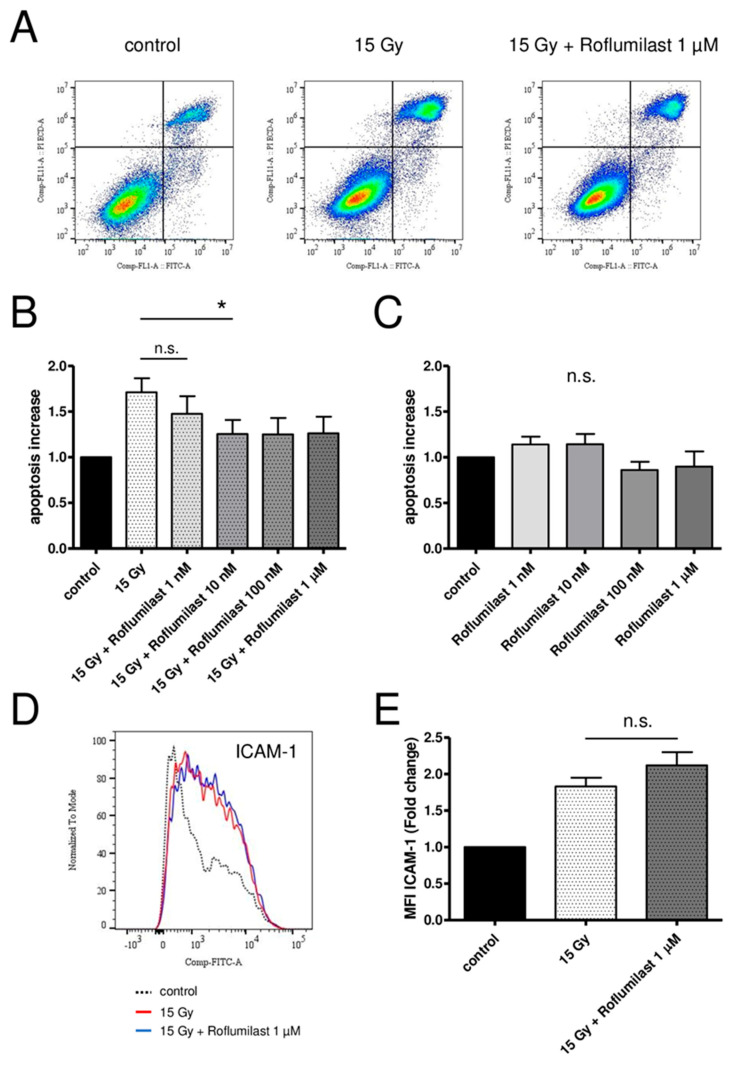
Effects of Roflumilast treatment on late apoptosis and ICAM-1 expression in control and high-dose-irradiated (15 Gy) HPMECs. (**A**) Analysis of apoptosis using flow cytometry in control HPMECs, after high-dose irradiation (15 Gy), and in irradiated HPMECs treated with Roflumilast (1 µM). All measurements were performed 72 h after irradiation. Quadrants drawn on pictures allows to measure the proportion of apoptotic cells (Annexin V+/PI+) among HPMECs populations. (**B**) Apoptosis after irradiation at a high dose (15 Gy) of HPMECs, and in irradiated HPMECs treated with Roflumilast (from 1 nM to 1 µM) after irradiation. Data on graph represent the average of seven experiments (* *p* < 0.05). (**C**) Basal apoptosis in control and Roflumilast-treated HPMECs. Data on graph represent the average of four experiments. (**D**) Histogram of ICAM-1 staining in control and 15 Gy-irradiated HPMECs, with or without Roflumilast (1 µM) treatment. (**E**) Normalized MFI values of ICAM-1 staining in irradiated HPMECs treated or not with Roflumilast (1 µM). Data on graph represent the average of four experiments (n.s.: not significant).

A FITC Annexin V/Propidium Iodide apoptosis detection kit was used to measure apoptosis in confluent HPMECs 72 h after irradiation. In accordance with previous reports [20,35], high-dose X-irradiation (15 Gy) induced late apoptosis in confluent HPMEC monolayers, as evidenced by the increased fraction of apoptotic cells (Annexin V+/PI+) (Figure 5A). Post-irradiation treatment of HPMECs with Roflumilast at concentrations ≥10 nM reduced irradiation-induced apoptosis, while no significant effect was observed at 1 nM Roflumilast (Figure 5A,B). In non-irradiated monolayers, Roflumilast did not significantly change the basal apoptosis (Figure 5C).

### 3.6. Effect of Roflumilast on Irradiation-Induced ICAM-1 Overexpression in HPMECs

Confluent HPMECs were irradiated at a high dose (15 Gy). After 24 h, the cells were detached and fixed, and the membrane expression levels of adhesion molecules (ICAM-1, VCAM-1, E-selectin, P-selectin) were measured using flow cytometry. As previously reported, the membrane expression level of ICAM-1 increased 24 h after irradiation (Figure 5D), while it remained unchanged for the other adhesion molecules [20,35]. Treatment with Roflumilast (1 µM) did not have a significant effect on irradiation-induced ICAM-1 membrane overexpression (Figure 5E).

## 4. Discussion

Efficient countermeasures protecting the endothelium from ionizing rays are needed to alleviate irradiation-induced organ damages. For this reason, here, we examined the effect of Roflumilast, a PDE4 inhibitor increasing cAMP levels, on a panel of irradiation-induced endothelial dysfunctions in human pulmonary microvascular endothelial cells.

Using impedance measurements in endothelial monolayers, we first demonstrate that Roflumilast rapidly and dose-dependently increases the impedance of confluent HPMEC monolayers, which is likely related to a reinforcement of the endothelial barrier. In confluent endothelial monolayers, such an effect is indeed likely explained by the strengthening/stabilization of endothelial junctions linked to the Roflumilast-induced cAMP rise, which can promote the formation of stable/linear junctions while preventing the formation of focal adherens junctions [10,11,12,21,22,23]. In non-irradiated cells, VE-Cadherin distribution was not significantly changed 24 h and 72 h after Roflumilast treatment, indicating that the Roflumilast-induced impedance increase is not linked to an upregulation of plasma membrane VE-Cadherin expression. Cortical F-Actin distribution was not modified in non-irradiated cells treated with Roflumilast and the proportion of cells with stress fibers was not decreased. Altogether, these observations suggest that the Roflumilast-induced impedance increase is not linked to significant cytoskeleton modification or VE-Cadherin distribution in non-irradiated cells. Instead, this could be explained by subtle ultrastructural changes in the intercellular contact zone induced by cAMP, as previously revealed by electron microscopy analysis in microvascular endothelial cells [23].

After exposure to X-rays, the observed slow decrease in the impedance of endothelial monolayers corresponds to progressive endothelial barrier disruption and may be related to the loss of apoptotic cells, to the weakening of endothelial junctions, and to the formation of gaps between endothelial cells of the monolayer [20,35]. From 24 to 72 h after irradiation, the rate of irradiation-induced impedance decrease was significantly reduced upon Roflumilast treatment, indicating that this compound protects the endothelial barrier from the deleterious effects of irradiation. In the time course of experiments, this effect of Roflumilast may be explained by its protective action against apoptosis-related loss of monolayer cells, by the reduction in intercellular gaps, and by the stabilization/strengthening of endothelial adherens junctions associated with its protective effects on the actin cytoskeleton.

Irradiation at a high dose (15 Gy) induced the formation of numerous actin stress fibers, a weakening of cortical actin, and a long-lasting alteration in the adherens junctions in HPMEC monolayers. In irradiated HPMEC monolayers treated with Roflumilast (≥100 nM), VE-Cadherin distribution was not significantly different from that of non-irradiated cells, indicating that Roflumilast efficiently protects adherens junctions made of VE-Cadherin from irradiation, or promotes the relocalization of VE-Cadherin at the plasma membrane. A redistribution of VE-Cadherin to cell–cell junctions after a cAMP increase has been previously described in endothelial cells [23,36]. In irradiated cells, Roflumilast treatment also promotes the recovery of cortical actin and reduces the formation of stress fibers. This is in accordance with previous reports showing that cAMP-elevating agents can promote the formation of circumferential actin while preventing the formation of stress fibers [10,11,12,22,23,36]. In our study, the tight correlations between VE-Cadherin distribution, cortical actin distribution, and the proportion of cells exhibiting stress fibers strongly suggest that the mechanisms involved in the protecting effects of Roflumilast for adherens junctions are linked to the reinforcement of the cortical actin ring and the reduced proportion of irradiated cells expressing long stress fibers. Therefore, after Roflumilast treatment of irradiated cells, the reduced formation of stress fibers together with the reinforcement of cortical actin may reduce tension on endothelial junctions, limiting cell contraction, VE-Cadherin delocalization and the formation of Gaps between cells of the monolayer. Altogether, these Roflumilast effects can in part explain the impedance stability observed in irradiated monolayers from 24 to 72 h after Roflumilast treatment [36].

After irradiation, the decreased expression of VE-Cadherin at cell–cell contacts has been shown to rely on both degradation of the protein by proteolytic enzyme such as ADAM10 or Calpain and on internalization of the protein toward intracellular compartments [5,15,18]. Therefore, in addition to its effect on the cytoskeleton, one cannot exclude that Roflumilast protects adherens junctions through an inhibition of the degradation or the internalization of VE-Cadherin. Finally, another possible explanation may be that Roflumilast stimulates VE-cadherin expression and localization at the plasma membrane after irradiation, as cyclic AMP can stimulate VE-Cadherin gene transcription through activation of the CREB transcription factor in pulmonary microvascular endothelial cells [24]. Therefore, the protecting effect of Roflumilast for adherens junction after irradiation may involve the conjunction of several distinct mechanisms, which needs further investigation.

In contrast to its protective effects on adherens junctions, Roflumilast remained without an effect on the ICAM-1 overexpression induced by high-dose irradiation. This result is in agreement with previous reports showing that agents increasing cAMP were without effect on ICAM-1 overexpression induced by an inflammatory cytokine (TNF-α) [37,38].

Altogether, the results from this in vitro study demonstrate the protective effect of Roflumilast against irradiation-induced endothelial dysfunctions (endothelial barrier disruption, apoptosis, adherens junction and actin cytoskeleton alterations) in a primary microvascular endothelial cell model, suggesting that this PDE4 inhibitor may alleviate irradiation-induced organ damages in case of exposure to high doses of radiation. However, in vivo studies need to be conducted to ascertain this hypothesis. Roflumilast has been used successfully to reduce the disturbed endothelial permeability in ischemia and sepsis animal models [27,32]. In humans, it has been approved and used for the treatment of chronic obstructive pulmonary disease or psoriasis [33,34]. Clinical studies revealed that Roflumilast is well tolerated and safe, but adverse effects have been described, including gastrointestinal disorders (such as diarrhea, nausea, weight loss) and neuropsychiatric disorders [39,40]. After exposure to high doses of radiation, the potent anti-inflammatory effects of Roflumilast may be beneficial to counteract excessive inflammation occurring in tissues [1,2,3,4,5,6,7,8,9,40]. However, in case of exposure to very high doses of ionizing rays, its rare gastrointestinal adverse effects may interfere with the radiation-induced gastrointestinal syndrome [40,41].

## 5. Conclusions

Overall, our results indicate that the PDE4 inhibitor Roflumilast, a medicine used in the treatment of severe chronic obstructive pulmonary disease or psoriasis, counteracts high-dose irradiation-induced endothelial dysfunctions and preserves the functional integrity of the microvascular endothelium [33,34]. Importantly, Roflumilast was efficient when applied after irradiation, suggesting that this medicine may be of interest for the treatment of irradiation-induced vascular and organ damages.

## Data Availability

The original contributions presented in this study are included in this article. Further inquiries can be directed to the corresponding author(s).
